# Trial for Malpractice

**Published:** 1855-02

**Authors:** 


*Trial for Malpractice.*—A friend just left our office who has recently been victimized in a suit for malpractice somewhat severely. We had intended to have given a detailed account of this suit, but have been prevented by circumstances. We shall venture now to give a brief account of the features of the case.

Some two years since Adolphus Gates, a German laborer, fell from the roof of a low house, fracturing his radius and otherwise injuring his wrist, the weight of the body being received upon the palm of the hand. He was attended for some months after the accident by Dr. John J. Haksteen, a respectable German practitioner of this city. It was proved upon the trial that Dr. H. was attentive to the case, and there was no evidence of any neglect on his part. The only question in the trial was whether the patient suffered through ignorance or incompetence on the part of his surgeon. The attempt was made by the prosecution to prove a simple dislocation of the wrist, with perhaps a transverse fracture of the radius. The wrist at the time of the trial, was anchylosed, with some dropping away of the hand from the ulna, but without any actual displacement. The splints used were produced. They were two long pieces of shingle extending from the elbow to the ends of the fingers, but not as wide as usually made. It was also proved that at the time of the first dressing considerable force was applied.

This is the sum of the laical evidence. No neglect or carelessness having been proved it remained with the professional evidence to pronounce upon the propriety of the treatment. And here, as is disgracefully common in trials for malpractice, the widest differences of opinion were expressed. It is not too much to say that there was, on the part of some of the witnesses, an evident anxiety to illustrate their own skill, by depreciating that of the unfortunate defendant.

The opinion of the greater part of the medical witnesses was, that the fracture was one within the joint, that the anchylosis was the result of inflammation and ossific deposit there, and finally that there was now, and had been, no dislocation of the joint. These facts, if clearly proved, would have been sufficient, with a candid jury, to acquit the defendant.

But this was not all the evidence. Several physicians on the stand testified to an opinion that the anchylosis was the result of a dislocation which had never been properly reduced. One young gentleman, of some four or five years experience, had met with at least four cases of dislocation of the wrist-joint. This fact should be put on record as showing a very remarkable coincidence in surgical experience. Let us see.

Dupuytren denied the possibility of such a dislocation.

Voillemier saw one case which he thought of sufficient importance to preserve, dissect, and illustrate by elaborate drawings and descriptions.

Another case is reported in the London Medical Gazette for 1843.

Three surgeons, of very large experience, have told us that they never saw a case.

The witness on the stand had four cases (or perhaps five, for he was not sure) in the first four years of his experience.

Such evidence as this it is which lowers the surgeon in the estimate of community. Certainly one, and probably both of the legal gentlemen employed in this case were familiar with the extreme infrequency of this dislocation, and governed themselves accordingly.

The jury rendered a verdict for the plaintiff of six hundred dollars, based upon the evidence that a dislocation of the wrist-joint was a common accident, easily reduced, and readily cured. In doing this they seem to have assumed that that portion of the evidence which went to prove the anchylosis the result of a fracture, was biased by a determination to swear the defendant through at any rate.

We have been thus free in our criticism of the evidence in this case, believing that the whole system of trials for malpractice is so radically wrong, that the defendant must be mulcted in every case where he is not sustained by the entire professional evidence. A single dissenting voice among the surgeons on the stand is enough to turn the scale in favor of the plaintiff, toward whom the sympathies of the jury invariably run.

In this case the evidence of a single man, contradicting all surgical experience, and evidently based on an egregious error in diagnosis, outweighed the opinions of older and better surgeons, and subjected a poor, hard-working and intelligent practitioner to a judgment and costs heavy enough to sweep away the greater portion of the small earnings of many years, spent in ill-requited toil among our foreign population. H.

